# Expression of EBV Encoded viral RNA 1, 2 and anti-inflammatory Cytokine (interleukin-10) in FFPE lymphoma specimens: a preliminary study for diagnostic implication in Pakistan

**DOI:** 10.1186/1746-1596-6-70

**Published:** 2011-07-27

**Authors:** Taimoor I Sheikh, Ishtiaq Qadri

**Affiliations:** 1 NUST Center of Virology and Immunology, National University of Sciences & Technology, Sector H-12 Islamabad 44000, Pakistan

## Abstract

**Background:**

Epstein Barr Virus (EBV) plays a significant role as a cofactor in the process of tumorigenesis and has consistently been associated with a variety of malignancies. EBV encoded RNAs (EBER1 and EBER2) are the most abundant viral transcripts in latently EBV-infected cells and their role in viral infection is still unclear. Formalin Fixed Paraffin Embedded (FFPE) tissues of surgically removed carcinoma biopsies are widely available form but have never been exploited for expressional studies previously in Pakistan. Immunohistochemistry (IHC) and *in situ *hybridization (ISH) in FFPE biopsy tissues remains the gold standard for proving EBV relationship in a histopathological lesion but their reagents associated limitations confines their reliability in some applications. Recently introduced targeted drug delivery systems induce viral lytic gene expression and therefore require more sensitive method to quantify viral as well as cellular gene expression.

**Methods:**

Eight (8) lymphoma samples were screened to detect the EBV genome. Qualitative and quantitative expression of EBV Encoded RNAs (EBER1, EBER2) and anti-inflammatory cytokine (interleukin-10) in FFPE EBV positive lymphoma tissue samples were then analysed by using Reverse transcriptase Polymerase Chain Reaction (RT-PCR) and Real Time Polymerase Chain Reaction (qRT-PCR), respectively.

**Results:**

In this study we have successfully quantified elevated expressional levels of both cellular and viral transcripts, namely EBER1, EBER2 and anti-inflammatory cytokine (IL-10) in the FFPE Burkitt's lymphoma (BL) specimens of Pakistani origin.

**Conclusions:**

These results indicate that FFPE samples may retain viral as well as cellular RNA expression information at detectable level. To our knowledge, this is first study which represents elevated expressional levels of EBER1, EBER2 and IL-10 in FFPE tissue samples of Burkitt's lymphoma in Pakistan. These observations will potentially improve current lacunas in clinical as well as diagnostic practices in Pakistan and can be further exploited to develop new strategies for studying cellular and/or viral gene expression.

## Background

Epstein-Barr virus (EBV) [1] is a ubiquitous human γ-herpes virus infecting more than 90% of the population worldwide [2] and plays a significant role as a cofactor in the process of tumorogenesis. It has consistently been associated with a variety of malignancies including endemic Burkitt's lymphoma (BL) [1,2], nasopharyngeal carcinoma [3,4], T-cell lymphoma, Pyothorax-associated or methotrexate-associated B-cell lymphoma, Primary effusion lymphoma, gastric carcinoma [5], EBV associated hemophagocytic syndrome and approximately 50% of Hodgkin's diseases. Moreover it has been associated with different types of lymphoproliferative diseases especially in immunocompromised patients [6]. In immunocompromised patients with impaired cell mediated immunity, acute EBV infection is associated with the development of lymphoproliferative disease, with mortality rates between 50-80%. In addition to that, recipients of solid organ transplants, the incidence of post transplant lymphoproliferative disease (PTLD) ranges from 1% to 15% [7]. Prevalence of EBV in Pakistani Burkitt's lymphoma patients is 80% which is significantly higher than in BL in North America [8].

EBV replicates in the epithelial cells of the mouth, tongue, salivary glands, and oral cavity and then it spread into the B-cells which are the main host cell type for its latent infection [9]. Upon entry into B cell, the EBV proteins are expressed in a cascade manner. Every EBV-transformed cell carries multiple extrachromosomal copies of the viral episome and constitutively expresses a limited set of viral gene products referred to as latent proteins, which comprise of: Six (6) EBV nuclear antigens (EBNAs 1, 2, 3A, 3B, 3C and -LP) [9-11], three latent membrane proteins (LMPs 1, 2A and 2B) and transcripts from the *Bam*HIA region of the viral genome namely BART transcripts [11,12]. In addition to the latent proteins, EBV-transformed cell also show abundant expression of the small, non-polyadenylated non-coding RNAs, EBER1 and EBER2. This pattern of latent EBV gene expression, which appears to be activated only in Burkitt's lymphoma, is referred to as 'latency I' [9-13]. Interleukin-10 (IL-10) an anti-inflammatory cytokine (also known as human Cytokine Synthesis Inhibitory Factor (CSIF)), is known to be an important regulator in cell transformation [14]. EBV^+ ^(positive) Burkitt's lymphoma (BL) cells express high level of IL-10 as compared to the EBV^- ^(negative) BL cell lines [15]. On the other hand, the expressional levels of IL-10 are extremely low and/or negligible in normal tumour cell lines [15,16]. Approximately 10 fold increased expression of the IL-10 in Burkitt's lymphoma cell lines is reported [16]. These elevated levels of EBER and IL-10 have also been known to increase cell's tumorigenicity [17,18]. This expression profile could be used as power full tool in the expressional profiling studies as well as relative quantification of EBERs in the tissue samples.

For tissue preservation, formalin fixation followed by paraffin wax embedding is mostly used to maintain the morphological features of the original tissue [19]. The Formalin Fixed and Paraffin Embedded (FFPE) tissues are used in various immunohistochemistry (IHC) techniques for localizing Epstein-Barr viral nucleic acid and/or protein to the tumour cells. Previously EBER in-situ hybridization (ISH) was considered as the most excellent test for detection and localization of latent EBV in tissue samples [20,21]. Other techniques including automated EBER ISH and automated LMP-1 IHC with increased sensitivity and specificity have recently became useful diagnosis tool for EBV related diseases [22-24].

ISH reagents associated limitations like limited target site accessibility and capacity to penetrate into whole mount tissue specimens confines its reliability [25]. Although, both techniques (fluorescent in-situ hybridization (FISH) and reverse transcriptase polymerase chain reaction (RT-PCR)) have inevitable advantages in the field of diagnostics but comparative evidence of more target specificity and sensitivity of RT-PCR for the detection of minimal residual disease in long-term monitoring of patients has increased its importance in some applications [26]. In addition to that quantitative real time PCR which has ability to detect as low as two fold changes in the gene expression, is rapidly replacing immunological assays [27,28]. Numerous therapeutic agents aiming EBV associated tumour regression are beginning to emerge [29,30]. These targeted drug delivery systems induce viral lytic gene expression [30,31] and therefore, require more sensitive quantitative analysis of viral as well as cellular gene expression to monitor the effect of these therapeutic treatments.

This study was aimed to develop a precise Real time PCR based laboratory methodology to detect and quantify EBV encoded RNAs in FFPE Burkit's lymphoma samples of Pakistani origin. Furthermore, anti-inflammatory cytokine (IL-10) expression in EBV^+ ^(positive) samples were characterized to indicate its relevance with viral persistence in tumour cells. These studies will introduce novel potential biomarkers as well as new insight in the current diagnostic trends of Pakistan.

## Methods

### Sample Collection and Cell Culture

In total eight (8) lymphoma samples were included in this study. The sampling was conducted at Pakistan Institute of Medical Sciences (PIMS), for routine histopathological examination. Formalin Fixation and Paraffin Embedding (FFPE) procedures were then carefully performed [32-35] on lymphoma samples individually, under sterile conditions. After histopathological examination three (3) samples were classified as Burkitt's lymphoma (BL) and five (5) as non-Hodgkin lymphomas (NHLs).

EBV transformed *B95-8 *cell line (Kindly provided by Dr. Sharof Tugizov Department of Medicine, University of California, San Francisco, California USA) were maintained in RPMI 1640 supplemented with 10% fetal bovine serum (FBS), 1% L-glutamine, and 1% penicillin-streptomycin (all from Gibco, Basel, Switzerland).

### Nucleic Acid Extraction and Purifications

Approximately 4 × 20 μm sections of selected samples were taken for nucleic acid extraction and purification. Genomic DNA was purified from Formalin-fixed Paraffin embedded tissue sections by using QIAamp DNA FFPE Tissue Kit (Qiagen).

Total RNA of the positive samples was extracted by using PureLink™ FFPE Total RNA Isolation Kit (Invitrogen Carlsbed, CA U.S.A) according to manufacturer instructions. All samples were treated with DNase I Amplification Grade (Invitrogen Carlsbed, CA U.S.A) to eliminate DNA contaminations.

### Amplification and Detection

Before EBV specific amplifications, all DNA samples were tested with primers to amplify a fragment of the human β globin Genes in order to ensure DNA integrity. Initially, nested PCR based screening for EBV presence of all lymphoma samples were carried out by targeting genomic DNA of EBNA1 as recommended by Bagan JV et al, 2008 [36].

Qualitative PCR of EBV positive samples were performed in total volume of 20 ul containing final concentrations of 100 ng genomic DNA, Taq Buffer with KCl; 2.5 units of Taq DNA polymerase, 2 mM dNTPs, 1.5 mM MgCl2 final concentration (Fermentas). In the first round of amplification, primers for EBER1 and EBER2 (Table 1) aimed at amplifying a fragment of 166 bp and 172 bp, respectively.

**Table 1:** Specification of Primers used in RT-PCR and qRT-PCR Experiments

**S. No**.	Target Gene	Primer Name	Primer Sequence 5'-----------------3'
1)	EBV encoded RNA 1 (EBER1)	EB1 (Forward) EB1 (Reverse)	5'- AGGACCTACGCTGCCCTAGA -3' 5'- AAAACATGCGGACCACCAGCTGG -3'

2)	EBV encoded RNA 2 (EBER2)	EB2 (Forward) EB2 (Reverse)	5'- AGGACAGCCGTTGCCCTAGTG -3' 5'- TAGCGGACAAGCCGAATACCCT -3'

3)	Interleukin 10 (IL10), mRNA	IL-10 (Forward) IL-10 (Reverse)	5'- ATGCACAGCTCAGCACTGCTCTG -3' 5'- GGAAGAAATCGATGACAGCGCCG -3'

4)	Glyceraldehyde-3-Phosphate Dehydrogenase (GAPDH), mRNA	GAP (Forward) GAP (Reverse)	5'- CAAGGTCATCCATGACAACTTTG -3' 5'- GTCCACCACCCTGTTGCTGTAG -3'

### Quantitative Real time PCR (qRT-PCR)

Quantitative Real time PCR (qRT-PCR) of 10 ul of Total RNA template (50 ng) of EBV^+ ^(positive) and EBV^- ^(negative) samples were used to amplify EBER 1/EBER 2 and IL-10 aimed primers (Table 1). Thermocycling was performed at 95°C for 3 minutes, followed by 40 cycles at 94°C for 30 s, 54°C for 30 s, and 72°C for 40 s to measure the fluorescence signal. Quantitative analyses of the data were carried out using 7300 system SDS software v1.4 (Applied Biosystems, USA). Reaction concentrations and conditions were adjusted according to the manual instructions of SuperScript^®^III Platinum^® ^SYBR^® ^Green One-Step qRT-PCR Kit (Invitrogen Carlsbed, CA, U.S.A). Equal quantity of RNA from *B95-8 *cell line (Positive control) and EBV^- ^tissue sample (Negative control) were used. All experiments were done in triplicate.

### Purification and Sequencing

The amplified product was purified by using PureLink™ Gel Extraction Kit (Invitrogen Carlsbed, CA U.S.A) according to the manufacturer's instructions. The purified product was sequenced by using Genetic Analysis System Beckman Coulter (CEQ-8000 USA). These sequences were published in Genbank, EMBL and DDBJ [Accession Number: GU205106 &GU205107].

### Date Analysis

In a real time PCR assay Ct (cycle threshold) is defined as the number of cycles required by fluorescent signals to cross the threshold. Ct levels are inversely proportional to the quantity of target nucleic acid in the sample [37,38].

In these experiments, the linear rates of expressions of EBER1, EBER2 and IL-10 were calculated by dividing the threshold (0.10) with respective mean CT values of EBV^+ ^patients.

## Results and Discussions

**Figure 1:**
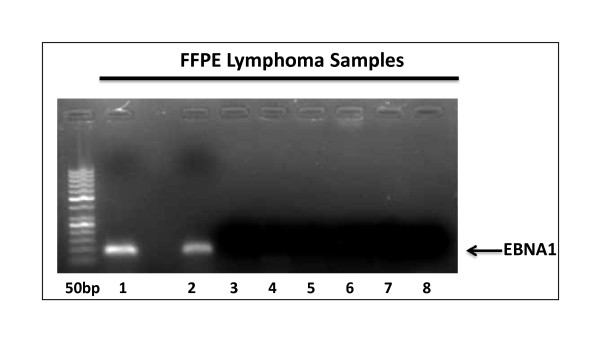
**Results of agarose Gel Electrophoresis of EBNA 1 genomic DNA (left-to-right)**: Lane 1 shows the 50 bp DNA ladder, Lane 2-3 represents the EBNA1 positive Burkitt's lymphoma samples (100 bp Nested PCR amplified product), Lane 4 represents EBNA1 Negative Burkitt's lymphoma (BL) samples, Lane 5,6,7,8 represents EBNA1 Negative non-Hodgkin lymphomas (NHLs) samples.

**Figure 2:**
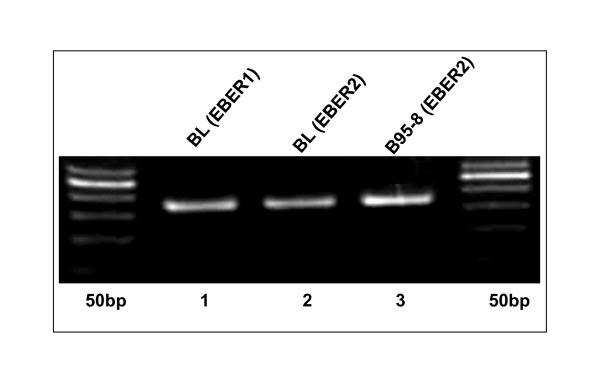
**Qualitative PCR assay system**: Results of Agarose Gel Electrophoresis of EBER1 and EBER2 genomic DNA fragment in EBV positive Burkitt's lymphoma (BL) samples. Lane 1 (left-to-right) 50 bp ladder, Lane 2 166 bp EBER1, Lane 3 172 bp EBER2, Lane 4 EBER 2 172 bp (B95-8), positive control, Lane 5 50 bp ladder.

Epstein Barr Virus (EBV) play significantly as a cofactor in the process of viral induced oncogenesis and associated with a larger range of malignancies [2-5]. Here, we have screened eight (8) FFPE lymphoma samples of for the presence of EBV nuclear antigen (EBNA1) (Figure 1). In addition to that, EBV encoded RNAs (EBER1 and EBER2) are the most abundant viral transcripts in latently EBV-infected cells [12,13] and their role in viral infection is still unclear. In this study, we have developed a qualitative reverse transcriptase (RT-PCR) based assay system (Figure 2) to detect genomic fragments of EBER1 and 2 from Formalin Fixed Paraffin Embedded (FFPE) tissue sample. The FFPE sections are most broadly available form of lymphoma tissues in Pakistan.

**Figure 3:**
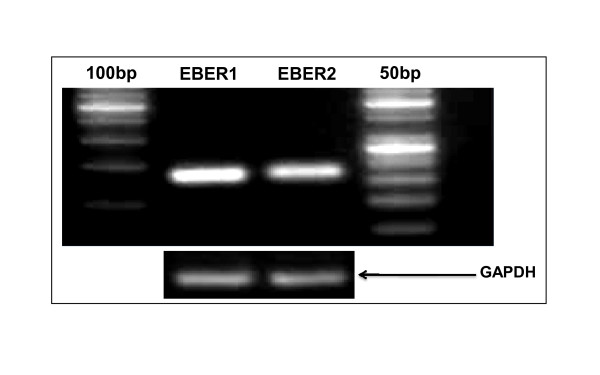
**Electropherogram showing semi-quantitative RT-PCR based analysis of EBER1 and EBER2 (left-to-right)**: Lane 1 (100 bp ladder), Lane 2 transcript of EBER1 (166 bp), Lane 3 transcript of EBER2 (172 bp), Lane 4 50 bp ladder. All samples were normalized with GAPDH as reference genes.

**Figure 4:**
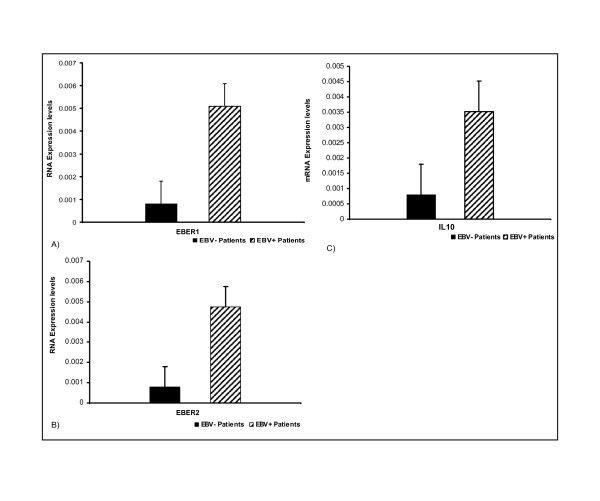
**Real-time PCR based Quantitative analysis of EBER1, EBER2 and IL-10 transcripts**: Real-time PCR was performed to determine the expression status of IL-10, EBER1 and EBER2. GAPDH was used as a reference gene to normalize expression of all samples. Expression levels of EBER1 (A), EBER2 (B) and IL-10(C) in EBV infected Burkitt's lymphoma samples were quantified and compared to uninfected samples as negative controls. Data are shown after log transformation.

**Figure 5:**
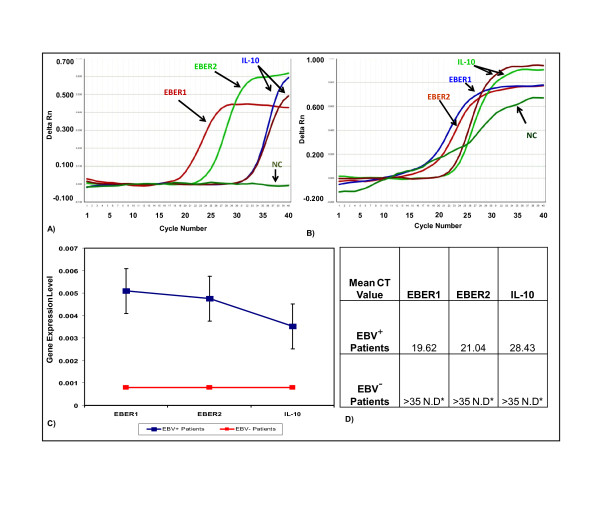
**qRT-PCR Data analysis**: Delta Rn vs Cycle (A, B): Real-time PCR amplification plot showing EBER1, EBER2 and IL-10 targets in EBV^+ ^(positive) tumor sample in comparison with EBV Negative control sample analyzed at 0.1 threshold. Successful amplifications are shown, with absence of amplification of mismatched templates. (C) Relative Expression rate of EBER1, EBER2 and IL-10 were calculated by dividing the total threshold (0.10) with mean CT value of each sample.(D) Grid representation of specificity showing mean CT values for on-target cDNA amplification for all EBV^+ ^(positive) specimens. Note the absence of nonspecific amplification. N.D*= Not Detected.

Viral gene expression based studies provide a better chance to understand virally induced lymphomas at molecular level which leads to the discoveries of potential biomarkers and therapeutic agents [39-41]. Several drugs involved in tumour degeneration require knowledge of change in gene expression in diseased tissues [30,31]. Furthermore, FFPE samples have not yet been analytically investigated with respect to viral gene expression in Pakistan. We have for the first time purified, amplified (Figure 3) and sequenced [Genbank, EMBL, DDBJ Accession Number: GU205106 &GU205107] viral RNAs from the FFPE Burkit's lymphoma tissue samples. These results positively suggest the use of FFPE samples for research, more prominently in viral RNA profiling studies. In addition to that, we have also developed a real time PCR based assay in which a novel comparison of viral and cellular RNA is illustrated (Figure 4). This was done on the bases of an observation that expression of IL-10 mRNA was negligibly low in normal EBV negative lymphoma tissues [14-17]. We have successfully quantified and reported elevated level of IL-10 mRNA and EBV encoded viral RNAs in comparison with normal EBV^- ^subjects (Figure 5). These observations can be further used to develop precise laboratory techniques with increased reliability.

## Conclusions

Expression of both viral RNA and cellular RNA are significantly high, in comparison with normal EBV^- ^(negative) lymphoma tissues. These results suggested that RNA isolated from FFPE samples preserve the majority of the characteristic expression. In addition to that elevated expression of anti-inflammatory cytokine (IL-10) was observed in the EBV^+ ^(positive) samples in comparison with EBV^- ^samples (undetectable or negligibly low levels of expression) which indicate its relevance with viral persistence in EBV transformed tumour cells. These studies will open novel avenues to study cellular and/or viral gene expression and will introduce new biomarkers and strategies to improve clinical as well as diagnostic practices in Pakistan.

## Competing interests

The authors declare that they have no competing interests.

## Authors' contributions

TIS have contributed to the conception, design, acquisition, analysis and interpretation of data and have been involved in drafting the manuscript as well as revising it critically for important intellectual content to be published and have given final approval of the version to be published.

IQ has contributed to the critical revision of important intellectual content and has given final approval of the version to be published as well as provided supervision during this work.
